# Penile cancer: a Brazilian consensus statement for low- and middle-income countries

**DOI:** 10.1007/s00432-020-03417-1

**Published:** 2020-10-26

**Authors:** Andrey Soares, Icaro Thiago de Carvalho, Aluízio Gonçalves da Fonseca, Antonio Machado Alencar, Carlos Heli Bezerra Leite, Diogo Assed Bastos, João Paulo Holanda Soares, Katia Ramos Moreira Leite, Mário Ronalsa Brandão Filho, Ronald Wagner Pereira Coelho, Sandro Roberto de A. Cavallero, Stênio de Cassio Zequi, José de Ribamar Rodrigues Calixto

**Affiliations:** 1 Department of Oncology, Centro Paulista de Oncologia-Oncoclínicas, Av. Brigadeiro Faria Lima, 4300, Vila Olímpia, São Paulo, SP 01452-000 Brazil; 2 Department of Oncology, Hospital Israelita Albert Einstein, Av. Albert Einstein, 627, Morumbi, São Paulo, SP 05652-900 Brazil; 3 Latin American Cooperative Oncology Group, Porto Alegre, Rio Grande do Sul Brazil; 4 Department of Radiotherapy, Hospital Israelita Albert Einstein, São Paulo, São Paulo Brazil; 5 Instituto Abathon, São Paulo, São Paulo Brazil; 6 Department of Urology, Hospital Ophir Loyola, Belém, Pará Brazil; 7 Department of Oncology, Hospital Universitário da Universidade Federal do Maranhão, São Luís, Maranhão Brazil; 8 Department of Oncology, Hospital São Domingos, São Luís, Maranhão Brazil; 9 Department of Radiotherapy, Hospital Haroldo Juaçaba, Fortaleza, Ceará Brazil; 10 Department of Oncology, Hospital Sírio-Libanês, São Paulo, São Paulo Brazil; 11 Department of Oncology, Hospital Haroldo Juaçaba, Fortaleza, Ceará Brazil; 12 Medical Research Laboratory of the Discipline of Urology, Faculdade de Medicina da USP, São Paulo, São Paulo Brazil; 13 Department of Urology, Santa Casa de Misericórdia de Maceió, Maceió, Alagoas Brazil; 14 Department of Oncology, Hospital do Câncer Aldenora Bello, São Luís, Maranhão Brazil; 15 Department of Oncology, Hospital Adventista de Belém, Belém, Pará Brazil; 16 Department of Oncology, Centro de Tratamento Do Pará, Belém, Pará Brazil; 17 Department of Urology, AC Camargo Cancer Center, São Paulo, São Paulo Brazil; 18 National Institute for Science and Technology in Oncogenomics and Therapeutic Innovation, AC Camargo Cancer Center, São Paulo, São Paulo Brazil; 19 Department of Urology, Hospital Universitário Presidente Dutra, UFMA, São Luís, Maranhão Brazil

**Keywords:** Penile cancer, HPV cancer-related, Cancer consensus, Urologic malignancy

## Abstract

**Purpose:**

Penile cancer is highly prevalent in low- and middle-income countries, with significant morbidity and mortality rates. The first Brazilian consensus provides support to improve penile cancer patients’ outcomes, based on expert’s opinion and evidence from medical literature.

**Methods:**

Fifty-one Brazilian experts (clinical oncologists, radiation oncologists, urologists, and pathologists) assembled and voted 104 multiple-choice questions, confronted the results with the literature, and ranked the levels of evidence.

**Results:**

Healthcare professionals need to deliver more effective communication about the risk factors for penile cancer. Staging and follow-up of patients include physical examination, computed tomography, and magnetic resonance imaging. Close monitoring is crucial, because most recurrences occur in the first 2–5 years. Lymph-node involvement is the most important predictive factor for survival, and management depends on the location (inguinal or pelvic) and the number of lymph nodes involved. Conservative treatment may be helpful in selected patients without compromising oncological outcomes; however, surgery yields the lowest rate of local recurrence.

**Conclusion:**

This consensus provides an essential decision-making orientation regarding this challenging disease.

**Electronic supplementary material:**

The online version of this article (10.1007/s00432-020-03417-1) contains supplementary material, which is available to authorized users.

## Introduction

Penile cancer is rare in developed countries, but the incidence tends to be higher in developing countries such as Africa, Asia, and South America (GLOBOCAN 2019). Brazil registered 5.7 cases per 100,000 persons-year between 1996 and 2006, and has the third highest incidence worldwide of penile cancer (Cardona and García-Perdomo 2017). More than 50% of penile cancer cases in Brazil occur in the North and Northeastern areas, confirming the relationship between penile cancer and low socioeconomic status (Favorito et al. 2008).

Penile cancer is highly aggressive (Razzaghi et al. 2018; Pham et al. 2017); thus, timely diagnosis and treatment are crucial. Patients’ low educational level and limited access to healthcare in low- and middle-income countries delay the diagnosis, resulting in patients receiving care in more advanced stages.

Actions to prevent, to simplify the early diagnosis, and to provide better management of penile cancer targeting the population and the health professionals, as well as high-quality clinical trials, are needed. Therefore, this consensus statement presents recommendations to improve medical care based on experts’ opinions and the best available evidence from the medical literature. The main audience for this consensus statement is clinical oncologists, urologists, radiation oncologists, and any other health professional involved in managing this disease.

## Methods

The first Brazilian Penile Cancer Consensus was held during the III International Symposium of Genitourinary Review on November 29th and 30th, 2019, in São Paulo, SP, Brazil, through an initiative of the Latin American Cooperative Oncology Group-Genitourinary section (LACOG-GU) and the support of the Brazilian Society of Clinical Oncology (SBOC), the Brazilian Society of Urology (SBU), the Brazilian Society of Radiotherapy (SBRT), and the Brazilian Society of Pathology (SBP).

The Brazilian Urology, Clinical Oncology, Radiation Oncology, and Pathology Societies selected 51 participants (25 clinical oncologists, 18 urologists, 5 radiation oncologists, and 3 pathologists) according to their recognized academic performance and extensive clinical experience.

The experts voted 104 multiple-choice questions during the meeting. An agreement ≥ 75% among the participants was considered a consensus. Otherwise, the most-voted answer was considered a recommendation. The option “abstention to vote” was available for those unable to choose an answer or with prohibitive conflicts of interest, and the vote was not counted. All answers considered ‘consensus’ or ‘recommendation’ were subjected to the best available evidence in the medical literature using Medline–PubMed and SciELO–Lilacs databases, and ranked according to a modified version of the Oxford Centre for Evidence-based Medicine–Levels of Evidence (CEBM 2020), as shown in Table 1.

**Table 1:** Definition of levels of evidence (modified from CEBM 2020)

Level of evidence	Characteristics
1a	Systematic review with homogeneity of randomized clinical studies
1a-	Systematic review with heterogeneity of randomized clinical studies
1b	Randomized clinical study
1b-	Non-randomized clinical study
2a	Systematic review with homogeneity of cohort studies
2a-	Systematic review with heterogeneity of cohort studies
2b	Individual cohort study
3a	Systematic review with homogeneity of case–control studies
3a-	Systematic review without homogeneity of case–control studies
3b	Individual case–control study
4	Case-series
5	Expert opinion

## Results

The main resolutions and recommendations of the First Brazilian Consensus on Penile Cancer are as follows. Additional file 1 contains all questions and voting results.

### Penile cancer risk factors

Neonatal circumcision, the education of men regarding proper hygiene habits, smoking reduction, human papillomavirus (HPV) vaccination, and other actions in the prevention of sexually transmitted diseases reduce the risk for penile cancer and should be encouraged (consensus).

Phimosis is related to penile cancer and should not be considered physiologic after the age of 6 years (Dillner et al. 2000). Circumcision of newborns, though not in adulthood (Larke et al. 2011), reduces the risk of penile cancer (LE: 3a), especially invasive penile cancer (Larke et al. 2011). Circumcision does, however, protect against penile HPV infection in adults, especially in HIV-positive patients (Yuan et al. 2019), and helps to maintain adequate genital hygiene, which is also essential in reducing the risk of malignancy (Frisch et al. 1995) (LE: 3b).

HPV infection is strongly related to penile cancer (Backes et al. 2009) (LE: 2a). Preventative actions, such as the use of condoms and HPV vaccination, are essential. HPV vaccination reduces the risk of penile cancer (LE: 1b), as it results in a significant decrease in genital, precancerous, and malignant lesions (Giuliano et al. 2011). However, the recommendations for HPV vaccination may vary between different countries.

Currently, in Brazil, the quadrivalent HPV vaccine (6, 11, 16, and 18) is available for girls aged 9–14 years, boys aged 11–14 years, men and women from 9 to 26 years living with HIV/AIDS, individuals undergoing transplants of solid organs or bone-marrow transplantation, and cancer patients.

The expansion of the age range for boys’ vaccination from the age of 9 years may better protect men and women, and reduce the spread of the HPV virus (recommendation, LE: 5) (Ministério da Saúde 2018). The HPV vaccination should not be considered mandatory for all cancer patients (consensus, LE: 5). The replacement of the quadrivalent HPV vaccine (6, 11, 16, and 18) to the nine-valent vaccine (6, 11, 16, 18, 31, 33, 45, 52, and 58) would not be advisable in terms of public health (consensus, LE: 2a), because the latter does not show superior cost-effectiveness results (Ng et al. 2018).

However, health professionals should carefully evaluate this information, as the cost-effectiveness studies that were available at the time of this consensus meeting were performed mainly in developed countries, where penile cancer has a low prevalence and drug costs differ significantly from developing countries.

Smoking is a direct, independent, dose-related risk factor for penile cancer (LE: 3b), as is the consumption of products derived from tobacco (Hellberg et al. 1987; Harish and Ravi 1995). Heavy smokers (more than ten cigarettes per day) have twice the risk compared with light smokers and non-smokers (Hellberg et al. 1987).

Educational campaigns for penile-lesion identification improve the early diagnosis of penile cancer and should be encouraged (consensus, LE: 5) as penile cancer has an easily recognizable slow-growing pattern. However, more than 50% of patients in Brazil present at an advanced stage at diagnosis (Favorito et al. 2008). Lack of knowledge is one of the main reasons that patients do not seek medical care earlier (Skeppner et al. 2012).
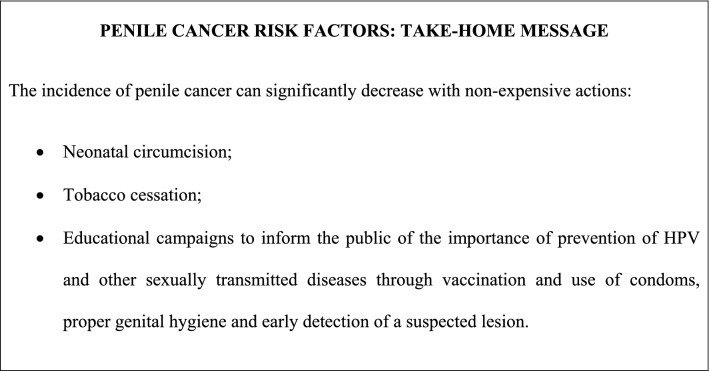


### Penile cancer staging

The recommendation for imaging examination in the staging of patients with penile SCC is chest computed tomography (CT) and abdominal and pelvic CT or magnetic resonance imaging (MRI) (recommendation, LE: 2b). CT helps to detect lymph-node involvement and systemic disease, with considerable sensitivity and high specificity in detecting metastatic lymph-node involvement (Zhu et al. 2008). MRI supports the investigation of local staging (Kayes et al. 2007). Bone metastases are uncommon and are usually associated with more advanced disease; therefore, patients should not routinely undergo bone scans unless they present signs and symptoms such as pain or increased alkaline phosphatase elevation (recommendation, LE: 4) (Braumann et al. 2015; Lal et al. 1999; Jacob et al. 1995).
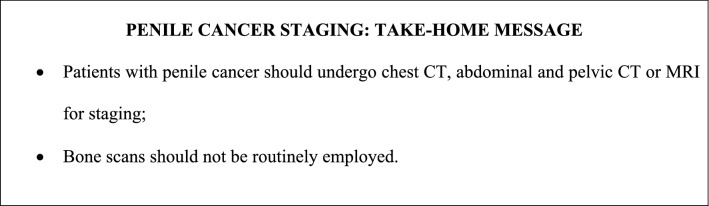


### Treatment of localized disease: primary tumor

Low-grade and low-stage penile carcinoma may benefit from organ-preserving penile procedures that can maintain normal appearance, organ function, and patient’s quality of life without compromising oncologic outcomes. Conservative treatment encompasses surgery, topical therapy, laser therapy, and radiation therapy.

Table 2 describes penile cancer staging according to the American Joint Committee on Cancer Tumor, Node, Metastases (AJCC TNM) guidelines and classification of the histopathological grading system by the World Health Organization and the International Society of Uropathology (WHO/ISUP) (Paner et al. 2018; Cubilla et al. 2018).

**Table 2:** Staging according to tumor-node-metastasis (TNM) classification and histopathological grading system (Paner et al. 2018; Cubilla et al. 2018)

*T: Primary tumor*		
Tis	Carcinoma in situ	
Ta	Non-invasive localized SCC, broadly extending invasion without destructive invasion	
T1	Tumor invades the subepithelial tissue layer in the glans, foreskin, or shaft regions	T1a: shows no lymphovascular invasion or perineural invasion and is not poorly differentiated
		T1b: shows lymphovascular invasion or perineural invasion or is poorly differentiated
T2	Tumor invades the corpus spongiosum	
T3	Tumor invades the corpus cavernosum	
T4	Tumor invades other adjacent structures	
*cN: Clinical loco-regional lymph nodes*		
cNx	Regional lymph nodes cannot be evaluated	
cN0	No visibly enlarged or altered inguinal lymph nodes on physical examination	
cN1	Palpable and mobile unilateral inguinal lymph nodes	
cN2	Bilateral or multiple mobile and palpable inguinal lymph nodes	
cN3	Palpable and fixed inguinal mass or unilateral or bilateral pelvic lymphadenopathy	
*pN: Pathological loco-regional lymph nodes*		
pNx	Regional lymph nodes cannot be evaluated	
pN0	No metastatic regional lymph nodes	
pN1	Up to two unilateral inguinal lymph-node metastases without extranodal extension	
pN2	More than two unilateral inguinal lymph-node metastases without extranodal extension	
pN3	Extra-capsular extension or unilateral or bilateral pelvic lymph nodes	
*M: Distant metastasis*		
M0	Absence of distant metastasis	
M1	Presence of distant metastasis	
*Stage*		
0	Tis or Ta, N0, M0	
1	T1a, N0, M0	
2	T1b or T2 or T3, N0, M0	
3	T1–3, N1, M0 or T1–3, N2, M0	
4	T4, any N, M0 or any T, N3, M0 or any T, any N, M1	
*Histopathological grading system*		
Gx	Histopathological grading cannot be evaluated	
G1	Well-differentiated	
G2	Moderately differentiated	
G3–4	Poorly differentiated/undifferentiated	

#### Tis and Ta tumors

The best conservative treatment of primary penile carcinoma in situ (Tis) is topical therapy (recommendation, LE: 2b). The use of topical 5-fluorouracil (5-FU) or imiquimod can provide from 40.0% to 73.7% complete response (Alnajjar et al. 2012; Lucky et al. 2015), with circumcision performed before therapy (Lucky et al. 2015). Patients presenting with non-invasive localized SCC (Ta) should be treated by partial glansectomy and resurfacing (recommendation, LE: 4). As most penile verrucous carcinomas are present in the glans, they have a slow progression and rarely present metastatically (Chuanyu et al. 2011; Li et al. 2015).

#### T1 tumors

Patients with T1aG1-2 should undergo glansectomy with grafting (recommendation, LE: 4), as this procedure can achieve 92% disease-specific survival (O’kane et al. 2011). The best conservative treatment of T1bG3 is glansectomy with reconstruction (recommendation, LE: 4), with which sexual function is preserved (Pietrzak et al. 2004).

Therefore, patients presenting with Tis, Ta, and T1 G1-3 disease may benefit from conservative treatment. However, they have a higher risk of local recurrence, and physicians must closely monitor these patients through follow-up, especially when presenting perineural invasion, Tis, positive definitive margins, and high-grade tumors (Albersen et al. 2018). Besides, organ-sparing surgery does not provide a significant difference in overall survival compared with total or partial penectomy (Lindner et al. 2019; Djajadiningrat et al. 2014a, b). In the case of local recurrence after conservative treatment, the best option is partial amputation (consensus, LE: 2b), which provides a high local-control rate (94%) (Lont et al. 2006), a low recurrence rate (approximately 5%), and the ability to maintain organ function (Leijte et al. 2008).

#### T2, T3, and T4 tumors

Partial amputation is also the best way to treat T2 disease when restricted to the corpus spongiosum/glans (consensus, LE: 2b) and T2/T3 invading the corpora cavernosa/urethra (recommendation, LE: 2b) (Ornellas et al. 2008). Partial amputation shows a significant 5-year local recurrence-free rate of approximately 60% (Lont et al. 2006), and 77% and 67% 5-year and 10-year survival rates, respectively (Kamel et al. 2018).

The best way to treat extended T4 tumors invading neighboring structures is total amputation/emasculation (recommendation, LE: 2b) (Ornellas et al. 2008). Neoadjuvant chemotherapy followed by surgery, if responsive, could be an option (recommendation, LE: 4), especially for patients with unresectable disease, yielding a 56% survival rate (Leijte et al. 2007a, b).

The best treatment for local recurrence after radical treatment of the primary lesion is total amputation (recommendation, LE: 2b), providing a less than 5% recurrence rate (Djajadiningrat et al. 2014a, b; Veeratterapillay et al. 2015).
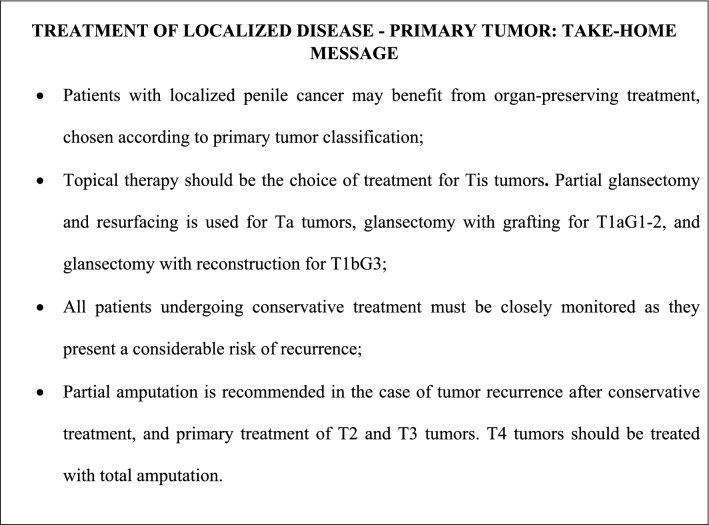


### Treatment of locally advanced disease: lymph-node involvement

Lymph-node metastasis is the most important independent predictor of survival in patients with penile carcinoma (da Costa et al. 2015; Hegarty et al. 2006; Horenblas and Tinteren 1994). The main prognostic factors for inguinal lymph-node metastases used to indicate lymphadenectomy are ≥ pT1b stage, microscopic lymphatic invasion, perineural invasion, invasion > 0.5 mm, and palpable lymph nodes after primary tumor resection and appropriate antibiotic therapy (consensus, LE: 2b) (Slaton et al. 2001; Ficarra et al. 2006; Velazquez et al. 2008).

Patients may present high risk or low risk of lymph-node involvement. High immunohistochemical expression of p53, MMP2, and MMP9, and low Ki-67 expression, the infiltrating growth pattern rather than growth by displacement (“pushing”) pattern, and unfavorable histology (usual type, basaloid, adenosquamous, and mixed and sarcomatoid squamous cell carcinoma) are variables associated with a higher risk of regional lymph-node metastases (Gunia et al. 2012; Zhu et al. 2007; Cubilla et al. 2001). These characteristics should be taken into account in the indication of lymphadenectomy whenever possible (recommendation, LE: 4). Intraepithelial neoplasms (Ta), pT1a stage, papillary or warty tumors, and tumors with non-invasive vertical exophytic growth are low-risk factors for inguinal lymph-node involvement that contribute to the contraindication for lymphadenectomy (consensus, LE: 2b) (Ornellas et al. 2008).

Healthcare professionals should consider early inguinal lymphadenectomy (2–6 weeks after primary tumor surgery), only for patients at high risk of lymph-node involvement (consensus, LE: 2b). This procedure significantly increases the 5- and 10-year survival rates in up to 91% of patients compared with 13% in cases of delayed lymphadenectomy (Kroon et al. 2005; McDougal 1995; Gulia et al. 2009; Ornellas et al. 2008). However, early lymphadenectomy is recommended in certain specific cases (recommendation, LE: 2b), such as in patients with uncertain adherence to the long-term follow-up needed after treatment of the primary tumor. This is because recurrence is possible up to 25 years after treatment, and early lymphadenectomy significantly improves patients’ survival (Ornellas et al. 2008).

Aspiration biopsy of suspected inguinal lymph nodes, guided by imaging methods, is a safe method for the investigation of lymph-node involvement. It may aid diagnosis, showing high sensitivity and specificity (87.3% and 99.9%, respectively) (Djajadiningrat and Teerst 2014), but it is not routinely recommended (recommendation, LE: 2b).

Radical inguinal lymphadenectomy should always be bilateral owing to presymphyseal lymphatic crossover (Park et al. 1994) and when imaging shows more than 50% bilateral inguinal drainage (Spiess et al. 2007) (consensus, LE: 4). An exception can be made when the sentinel lymph-node dynamic biopsy (BDLS) is negative on one side (recommendation, LE: 4). In such cases, patients must rigorously attend follow-ups, as there is a risk of occult micrometastasis in patients with negative BDLS (Spiess et al. 2007). The BDLS should be performed only at referral centers with trained staff (consensus, LE: 2b), as it may present a high rate of false-negative results (Leijte et al. 2007a).

Patients with only one affected inguinal lymph node without extra-capsular extension and size < 4.0 cm have a low risk of pelvic involvement (Lont et al. 2007); therefore, they should follow active surveillance and no other surgical procedure (recommendation, LE: 2b). Inguinal lymph-node dissection should be the next step for patients with unilateral mobile lymph-node enlargement ≥ 4 cm, and biopsy-confirmed lymph-node metastatic disease (recommendation, LE: 5). Pelvic lymphadenectomy is always indicated in the case of two or more affected inguinal lymph nodes or in the presence of extra-capsular extension (consensus, LE: 2b), as the risk of pelvic lymph-node involvement is significant (Lont et al. 2007; Zargar-Shoshtari et al. 2015; Ravi 1993). Patients with resectable pelvic lymph-node enlargement, identified on imaging during staging, should also undergo pelvic lymphadenectomy (consensus, LE: 2b). Unilateral inguinal lymphadenectomy is an acceptable recommendation, supported by low evidence publication (recommendation, LE: 4) (Tsaur et al. 2015). Bilateral pelvic lymph-node dissection (PLND) is always the first choice in patients with demonstrable unilateral metastatic disease as it shows more prolonged overall survival versus ipsilateral PLND (Zargar-Shoshtari et al. 2016).

The best immediate approach for patients with bilateral or fixed inguinal lymph-node enlargement without pelvic lymph-node enlargement, but also for those with fixed, stony or unresectable pelvic lymph-node enlargement identified on imaging, is neoadjuvant chemotherapy (conversion chemotherapy) (consensus, LE:1b-), with a cisplatin-based triple scheme (with taxane) (consensus, LE: 1b-). This approach shows statistically significant improvement in median progression-free survival and overall survival (Pagliaro et al. 2010; Xu et al. 2019), with a 50.0% response rate and 36.7% long-term progression-free survival (Pagliaro et al. 2010). For non-responsive patients, the palliative approach should be inguinal lymphadenectomy and radiotherapy (recommendation, LE: 1b-).

Patients with unilateral mobile lymph-node enlargement ≥ 4 cm undergoing neoadjuvant chemotherapy who have residual disease in one or more inguinal lymph nodes should undergo pelvic lymphadenectomy (consensus, LE: 5), as the chance of pelvic lymph-node involvement is high (Lont et al. 2007; Zargar-Shoshtari et al. 2015; Ravi 1993). For patients with resectable cN3 disease who lack a complete or partial clinical response to neoadjuvant chemotherapy, but show no disease progression during chemotherapy, the next approach should be primary tumor resection and inguinal and pelvic lymphadenectomy (consensus, LE: 2b). Patients with cN2 or cN3 disease with clinical response to neoadjuvant chemotherapy with ypN and residual disease should receive radiotherapy as adjuvant treatment (recommendation, LE: 2b) (Xu et al. 2019).

To evaluate the treatment response, to avoid continuing chemotherapy if the patient is not responding, or to change the planned treatment if the disease progresses, upper abdomen and chest CT should be used for distance disease assessment during or after neoadjuvant chemotherapy (consensus, LE: 5). MRI or pelvic CT is the most suitable imaging modality for loco-regional response evaluation of neoadjuvant chemotherapy (recommendation, LE: 5), as it can show deep lymph nodes and masses in the pelvis and retroperitoneum (Mao et al. 2014).

Patients with pN1 disease who have undergone primary tumor resection and inguinal lymphadenectomy without neoadjuvant treatment should receive only chemotherapy as adjuvant treatment (recommendation, LE: 4) as it prevents disease progression and improves 5-year overall survival (Pizzocaro et al. 1997). Combination with radiotherapy may only benefit patients with extensive disease (Choo et al. 2019). Patients not undergoing neoadjuvant treatment, but who have inguinal and pelvic lymphadenectomy and metastases in pelvic lymph nodes with bilateral inguinal lymph-node involvement or extranodal extension, should receive adjuvant treatment with chemotherapy and radiotherapy (recommendation, LE: 2b) with a cisplatin-based triple scheme (with taxane) (recommendation, LE: 2b), even if the evidence is still controversial. Adjuvant treatment improves overall survival (Sharma et al. 2015), but also shows no difference compared with no treatment (Kim et al. 2018; Choo et al. 2019).
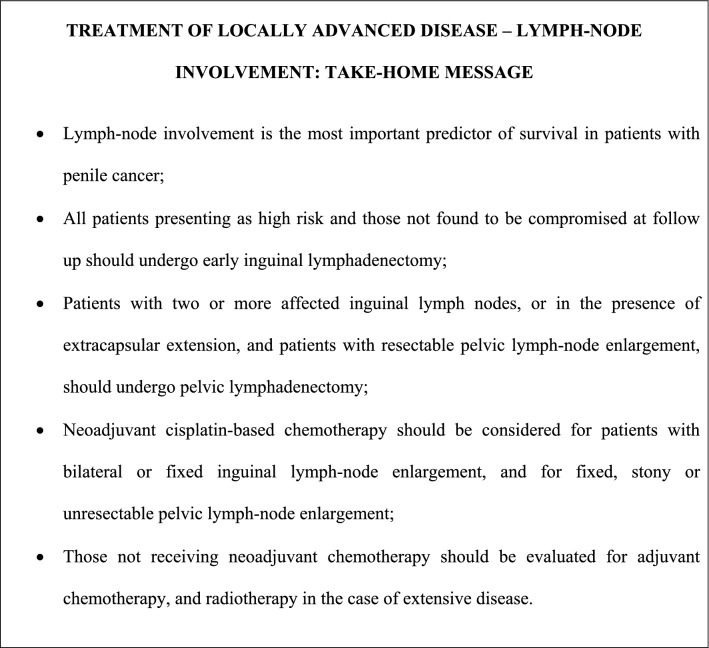


### Unresectable or relapsed tumors

The recommendation for patients with local recurrence infiltrating perineum without lymph-node involvement is neoadjuvant chemotherapy and surgical re-approach whenever possible (recommendation, LE: 4). This approach should also be considered for patients with advanced local recurrence and a unilateral or bilateral inguinal lymph-node mass, with or without vascular involvement (consensus, LE: 4). Chemotherapy yields a 60% rate of downstaging for unresectable tumors, and surgery on responders increases their 5-year overall survival by 32% (Leijte et al. 2007a, b).

Patients with local recurrence after chemotherapy/radiotherapy treatment without a prior surgical approach should undergo a surgical approach with the possibility of colostomy and cystostomy (consensus, LE: 5).

In first-line therapy for unresectable, recurrent and/or metastatic penile cancer, the standard therapy is systemic combination chemotherapy with cisplatin, ifosfamide, and paclitaxel (TIP) (recommendation, LE: 1b-), as well as other combinations of cisplatin, such as irinotecan, docetaxel, and fluorouracil (Nicholson et al. 2013; Theodore et al. 2008).
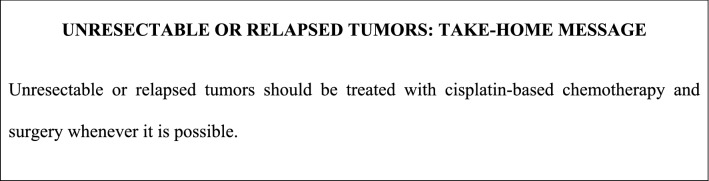


### Relapsed disease without the possibility of local rescue/metastatic disease

Systemic progression in penile cancer is beyond cure. Patients presenting with visceral metastasis should receive definitive palliative chemotherapy, whether they have a local recurrence or advanced tumors with a unilateral or bilateral fixed inguinal lymph-node mass, with or without vascular involvement but with visceral metastasis, or locally treated and controlled disease, but with visceral recurrence, or visceral recurrence after local surgical resection, chemo- and/or radiotherapy (consensus, LE: 1b-) (Nicholson et al. 2013; Theodore et al. 2008). The response rate of chemotherapy in advanced disease is low (Hakenberg et al. 2006), and a partial response may be achieved in a very limited number of patients (Haas et al. 1999; Pizzocaro et al. 2009). Patients presenting with local and visceral recurrence after surgical resection, first- and second-line chemotherapy, and/or radiotherapy should receive palliative care (consensus, LE: 5).

Currently, the role of anti-epidermal growth factor receptor (anti-EGFR) targeted therapy (cetuximab, panitumumab, and dacomitinib) in the treatment of advanced penile cancer is investigational (recommendation, LE: 1b-) as the available literature is still limited (Necchi et al. 2018; Carthon et al. 2014; Brown et al. 2014; Buonerba et al. 2016). Anti-EGFR is an option in unresectable, relapsed, and/or metastatic cancer as a second-line treatment, associated or not associated with chemotherapy, after TIP/TPF failure (recommendation, LE: 4), showing a trend to a higher response rate compared with other chemotherapy regimens (Buonerba et al. 2016).
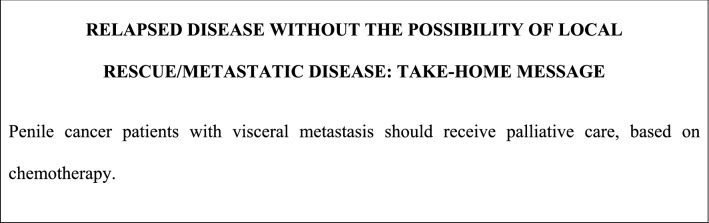


### Surgical aspects

Multimodal therapy is a good option for the treatment of advanced disease in penile cancer. The best approach after chemotherapy is surgery (consensus, LE: 2a-), providing a better chance for more extensive organ preservation by reducing the tumor mass. Compared with brachytherapy, surgery yields a lower overall recurrence rate (Hasan et al. 2015). However, surgeons should not consider hemipelvectomy and/or hemicorporectomy (recommendation, LE: 5).

The best surgical treatment option for urinary-tract reconstruction in disseminated metastatic disease is cystostomy (recommendation, LE: 5). Surgeons must avoid an ileal conduit for urinary-tract reconstruction/diversion in patients receiving multimodal therapy (recommendation, LE: 5). In locally unresectable disease with involvement of the posterior urethra and/or prostate, perineal urethrostomy should also be avoided as a means of urinary diversion (consensus, LE: 2b) owing to the high risk of stenosis after radiotherapy (Myers et al. 2011).

Chemoradiotherapy can provide adequate local control for resectable tumors that failed surgical resection (Langsenlehner et al. 2008) (recommendation, LE: 2b).
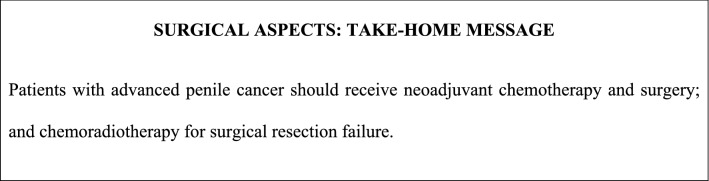


### Radiotherapy

SCC penile carcinoma is a radiosensitive tumor, enabling radiotherapy to provide treatment and organ preservation. Radiotherapy is indicated in case of patient preference and/or inoperability for comorbidities, in T1 or T2 tumors, in tumors less than 4 cm (recommendation, LE: 2b), with significant local control, and up to 87% of cases with organ preservation (Azrif et al. 2006; Crook et al. 2007; Cordoba et al. 2016). Such patients should undergo prostectomy before radiotherapy whenever possible (consensus, LE: 5), making the tumor more visually evident, avoiding complications, such as necrosis.

Radiotherapy can be the primary treatment modality in patients with small tumors (less than 4 cm), whereas surgery is a salvage procedure. Contact brachytherapy is used in patients presenting with T1 G1-2 tumors smaller than 4 cm (consensus, LE: 4), as a penile-preservation technique with significant tumor control and organ preservation (Marban-Orejas et al. 2020). Interstitial brachytherapy (consensus, LE: 2a-) treats T1 G1 to G4 and T2 tumors smaller than 4 cm. This technique presents an 83% rate of local control, with a 5-year local, regional recurrence-free survival (Rouscoff et al. 2014; Crook et al. 2010), and 5-year overall and disease-free survival similar to penectomy (Hasan et al. 2015). Teletherapy is also an option for radiotherapy (consensus, LE: 2b), with a 62% 5-year local control rate and a 40% rate for penile-preservation (Azrif et al. 2006).

Elective irradiation of inguinal and pelvic lymph nodes is never indicated (consensus for T1 G1-2; recommendation for T1 G3-4 and T2, LE: 2a), because surgery has superior benefits in terms of oncological outcomes in the case of lymph-node involvement, especially in high-grade tumors, and radiotherapy does not improve recurrence or survival rates (Robinson et al. 2018).

Patients with T1 G3-4 or T2 tumors smaller than 4 cm receiving radical radiotherapy should also receive chemotherapy (recommendation, LE: 4), thereby improving the chances of disease control (Chhabra et al. 2014). It is never indicated for patients with T1 G1-2 tumors smaller than 4 cm (consensus, LE: 5), as the evidence for a benefit in penile cancer is not available and limited in other cancer sites (Shylasree et al. 2011).

Patients with T1–T2 tumors larger than 4 cm undergoing radical radiotherapy should always receive elective irradiation of inguinal and pelvic lymph nodes, in concomitance with chemotherapy (recommendation, LE: 2b). This prevents extranodal extension and disease recurrence (Ravi 1993). Teletherapy is an acceptable radical-purpose radiotherapy technique (recommendation, LE: 5), but patients with tumors that size should not undergo interstitial brachytherapy (recommendation, LE: 2b) as the risk of recurrence is high (de Crevoisier et al. 2009). This technique can be used to increment the doses of teletherapy (recommendation, LE: 5).

Patients with T3–T4 tumors who receive radical-purpose radiotherapy should also receive elective irradiation of inguinal and pelvic lymph nodes (recommendation, LE: 2b), delivered concomitantly with chemotherapy (consensus, LE: 2b). Radiation therapy alone provides a low local-control rate (approximately 40%) and only a 38% penile-preservation rate (Zouhair et al. 2001). Interstitial brachytherapy is not an acceptable technique for such patients (consensus, LE: 5), nor is teletherapy (recommendation, LE: 5) or teletherapy with dose-enhanced interstitial brachytherapy (recommendation, LE: 5).

Adjuvant radiotherapy is indicated for patients with a compromised surgical margin (recommendation, LE: 5), and for those with cN2 disease not receiving neoadjuvant treatment and undergoing inguinal and pelvic lymph-node dissection, if ≥ pN2 (recommendation, LE: 2b). Adjuvant radiotherapy improves overall survival in patients with advanced disease with lymph-node involvement (Tang et al. 2017; Winters et al. 2018) and decreases recurrence rates (Tang et al. 2017). pT3–pT4N0 with free margins is not an indication for adjuvant radiotherapy in penile cancer (consensus, LE: 2b) as it does not improve oncological outcomes compared with surgery alone (Burt et al. 2014).

Conformational radiotherapy (RT3D) is the minimum technique for administering the radiation dose when radical teletherapy is indicated (with or without chemotherapy) in which lymph nodes will (consensus, LE: 2b) or will not be treated (recommendation, LE: 2b). The ideal technique is modulated intensity radiotherapy (IMRT) combined with imaging-guided radiotherapy (IGRT), based on the evaluation of IMRT from prostate cancer patients, with lower toxicity in pelvic organs compared with RT3D (Viani et al. 2019) (recommendation, LE: 2b).
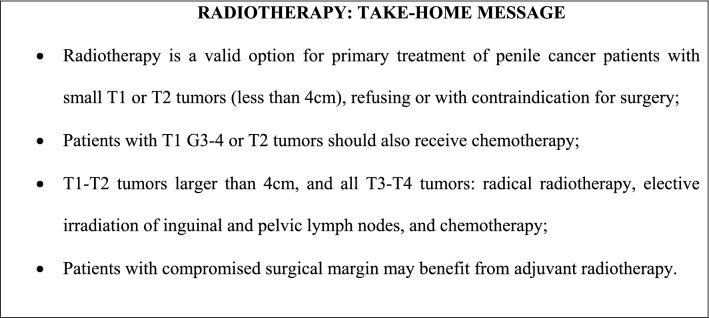


### Follow-up

Penile cancer has a significant impact on patients’ quality of life, including social, psychological, and sexual aspects (Drager et al. 2017; Ficarra et al. 2000). Therefore, psychological support should always be offered to patients after diagnosis (consensus, LE: 2b).

More than 90% of penile cancer recurrence occurs within the first 5 years after primary tumor treatment, especially during the first 2 years (Horenblas et al. 1993; Leijte et al. 2008). Follow-up of asymptomatic patients consists of anamnesis, clinical examination, and imaging every 3 months for the first 2 years, and every 6 months until the fifth year (recommendation, LE: 2b). The choice of imaging method depends on lymph-node involvement. Patients treated for early penile cancer (N0) are evaluated by US and Rx (recommendation, LE: 4) (Yamashita and Ogawa 1989), and patients with N1–N3 disease by CT and MRI (recommendation for N1, LE: 4; consensus for N2–3, LE: 4) (de Kerviler et al. 1995).

The follow-up of patients with advanced penile cancer (unresectable/inoperable) should be established individually, with referral to palliative care as needed (consensus, LE: 5).
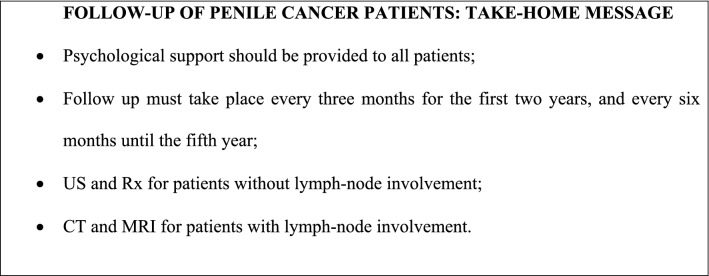


### Bone therapy

Bone is a rare distant metastasis site for penile cancer, and the evidence for management is low. Clinical decisions are made based on extrapolation of data from other solid tumors with a high incidence of bone metastasis, such as prostate cancer.

Bone-modifying agents should be indicated for all penile cancer patients with bone metastasis (without clinical contraindications, e.g., allergies, kidney failure, or others) (consensus, LE: 5), as it decreases or delays skeleton-related events (Saad et al. 2004). There is no preference among the bone-modifying agents (recommendation, LE: 5). Even if no comparative study in penile cancer patients is available, denosumab shows a trend toward superiority (Fizazi et al. 2011). The recommended dose and frequency of zoledronic acid are 4 mg IV every 4 weeks (recommendation, LE: 5). For patients using denosumab, 120 mg SC should be administered every 4 weeks (consensus, LE: 5). Therapy with bone-modifying agents is used without a limited duration or until a significant and/or intolerable adverse event occurs (consensus, LE: 5).
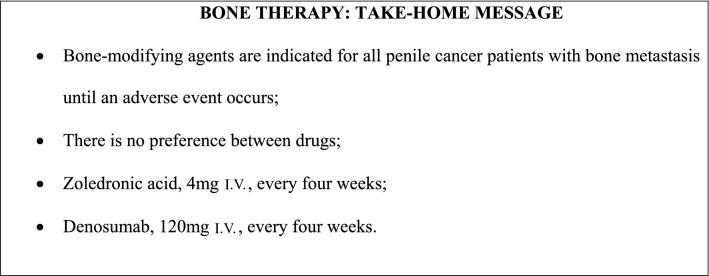


## Conclusion

Penile carcinoma is a challenging disease, with a high incidence in low- and middle-income countries, with limited health system resources and populations with low education hampering more efficient management. This consensus statement delivers an important decision-making orientation regarding penile carcinoma, in which improvement of care is particularly required. The evidence levels are generally low owing to the low incidence of the disease in developed countries and, consequently, the lack of large, randomized investigations. More clinical studies are needed, and whenever possible, specialists should refer patients to clinical trials.

## Electronic supplementary material

Below is the link to the electronic supplementary material.

Supplementary file1 (DOCX 45 kb)
