# Supplementary material for: Penile cancer: a Brazilian consensus statement for low- and middle-income countries
Source: J Cancer Res Clin Oncol. 2020 Oct 26;146(12):3281–96. doi: 10.1007/s00432-020-03417-1 (PMC7679332; doi:10.1007/s00432-020-03417-1)
Supplement: Supplementary file 1 — Supplementary file1 (DOCX 45 kb) [file 432_2020_3417_MOESM1_ESM.docx]

**SUPPLEMENTARY MATERIAL:** Voting results from expert meeting regarding management of penile cancer.

**Journal of Cancer Research and Clinical Oncology**

**Penile cancer: a Brazilian consensus statement for low- and middle-income countries**

**Authors:** Andrey Soares^1,2,3^, Icaro Thiago de Carvalho^4,5^, Aluízio Gonçalves da Fonseca^6^, Antonio Machado Alencar Jr.^7,8^, Carlos Heli Bezerra Leite^9^, Diogo Assed Bastos^3,10^, João Paulo Holanda Soares^11^, Katia Ramos Moreira Leite^12^, Mário Ronalsa Brandão Filho^13^, Ronald Wagner Pereira Coelho^3,14^, Sandro Roberto de A. Cavallero^3,15,16^, Stênio de Cassio Zequi^17,18^, José de Ribamar Rodrigues Calixto^19^

**ORCID:**

0000-0003-4980-6729 (Soares A)

0000-0003-2161-0222 (Leite C)

0000-0002-2615-7730 (Leite K)

0000-0003-0607-7589 (Carvalho IT)

**Affiliations:**

1. Department of Oncology, Centro Paulista de Oncologia-Oncoclínicas, São Paulo, São Paulo, Brazil

2. Department of Oncology, Hospital Israelita Albert Einstein, São Paulo, São Paulo, Brazil

3. Latin American Cooperative Oncology Group, Porto Alegre, Rio Grande do Sul, Brazil.

4. Department of Radiotherapy, Hospital Israelita Albert Einstein, São Paulo, São Paulo, Brazil

5. Instituto Abathon, São Paulo, São Paulo, Brazil

6. Department of Urology, Hospital Ophir Loyola, Belém, Pará, Brazil

7. Department of Oncology, Hospital Universitário da Universidade Federal do Maranhão, São Luis, Maranhão, Brazil.

8. Department of Oncology, Hospital São Domingos, São Luis, Maranhão, Brazil.

9. Department of Radiotherapy, Hospital Haroldo Juaçaba, Fortaleza, Ceará, Brazil.

10. Department of Oncology, Hospital Sírio-Libanês, São Paulo, São Paulo, Brazil

11. Department of Oncology, Hospital Haroldo Juaçaba, Fortaleza, Ceará, Brazil.

12. Medical Research Laboratory of the Discipline of Urology, Faculdade de Medicina da Universidade de São Paulo, São Paulo, São Paulo, Brazil.

13. Department of Urology, Santa Casa de Misericórdia de Maceió, Maceió, Alagoas, Brazil.

14. Department of Oncology, Hospital do Câncer Aldenora Bello, São Luís, Maranhão, Brazil

15. Department of Oncology, Hospital Adventista de Belém, Belém, Pará, Brazil.

16. Department of Oncology, Centro de Tratamento do Pará, Belém, Pará, Brazil.

17. Department of Urology, AC Camargo Cancer Center, São Paulo, São Paulo, Brazil.

18. National Institute for Science and Technology in Oncogenomics and Therapeutic Innovation, AC Camargo Cancer Center, São Paulo, São Paulo, Brazil

19. Department of Urology, Hospital Universitário Presidente Dutra, UFMA, São Luís, Maranhão, Brazil.

**Corresponding author:**

Andrey Soares, MD

Division of Oncology, Hospital Israelita Albert Einstein. Av. Albert Einstein, 627 - Morumbi, São Paulo/SP - CEP: 05652-900, Brazil

Division of Oncology, Centro Paulista de Oncologia. Av. Brigadeiro Faria Lima, 4300 - Vila Olímpia, São Paulo/SP - CEP: 01452-000, Brazil

Phone: +55 (11) 98315-6449

E-mail: [dr.andrey@uol.com.br](mailto:dr.andrey@uol.com.br)

The consensus meeting was held on November 29th and 30th, 2019, in São Paulo, SP, Brazil.

Fifth-one participants voted the 104 multiple-choice questions below. The results were rounded up to the next higher number if the decimal number was between 5 and 9, or down to the next lowest number if it was between 1 and 4. The option “abstention” was available in each question, and the percentage of participants choosing that alternative was not considered. For each question, the answer reaching at least 75% of the voters’ opinion was considered a consensus and presented in this paper. If none of the answers reached ≥75%, the most voted answer was considered a recommendation, and chosen to be discussed in this paper.

# **RISK FACTORS and PENIle CANCER staging**

1. Circumcision in neonates reduces risk for penile cancer and should be stimulated:

a. Yes 80%

b. No 20%

c. Abstention

2. Smoking reduction reduces risk for penile cancer and should be encouraged:

a. Yes 91%

b. No 9%

c. Abstention

3. Education about proper hygiene habits reduces the risk for penile cancer and should be encouraged:

a. Yes 100%

b. No 0%

c. Abstention

4. Preventing sexually transmitted diseases and encouraging safer sex reduces the risk of penile cancer and should be encouraged:

a. Yes 100%

b. No 0%

c. Abstention

5. HPV vaccination reduces risk for penile cancer and should be encouraged:

a. Yes 92%

b. No 8%

c. Abstention

6. Educational campaign on penile lesion recognition improves early diagnosis for penile cancer and should be encouraged:

a. Yes 98%

b. No 2%

c. Abstention

7. The quadrivalent HPV vaccine (6, 11, 16 and 18) is available for girls aged 9 to 14, boys aged 11 to 14, HIV carriers 9 to 26 years, and immunocompromised (solid organ transplantation, bone marrow transplantation and cancer patients). Should the age range for boys be expanded?

a. Yes 59%

b. No 41%

c. Abstention

8. Should HPV vaccination be considered mandatory for cancer patients?

a. Yes 13%

b. No 87%

c. Abstention

9. Would it be advisable for public health to change the HPV vaccine (6, 11,16 and 18) from quadrivalent to nonavalent (6,11,16,18,31,33,45,52,58)?

a. Yes 22%

b. No 78%

c. Abstention

10. What is the recommendation for imaging examination in staging of patients with squamous penile carcinoma (SPC)?

a. Complete abdomen ultrasound + chest x-ray 11%

b. Chest CT + MRI or CT of the abdomen and pelvis 15%

c. Chest CT + MRI or abdominal and pelvic CT + bone scan only if bone symptoms and/or increased alkaline phosphatase 57%

d. Chest CT + MRI or CT of the abdomen and pelvis + bone scan 6%

e. There is no indication 11%

f. Abstention

# **treatment of localized disease**

11. What is the best conservative treatment of primary lesion Tis?

a. Topical therapy 46%

b. Partial glansectomy and resurfacing 32%

c. Total glansectomy and resurfacing 4%

d. Laser therapy 11%

e. Total glansectomy 7%

f. Abstention

12. What is the best conservative treatment of primary lesion Ta?

a. Partial glansectomy and resurfacing 62%

b. Total glansectomy and resurfacing 10%

c. Laser therapy 17%

d. Total glansectomy 4%

e. External radiotherapy 0%

f. Brachytherapy 7%

g. Abstention

13. What is the best conservative treatment of primary lesion T1aG1-2?

a. Laser therapy 12%

b. Glansectomy with graft 47%

c. Glansectomy without graft 16%

d. Partial amputation 16%

e. External radiotherapy 0%

f. Brachytherapy 9%

g. Abstention

14. What is the best conservative treatment of primary lesion T1bG3?

a. Local excision + non-graft reconstruction 10%

b. Local excision + graft reconstruction 10%

c. Glansectomy with reconstruction 70%

d. Laser therapy 0%

e. External radiotherapy 0%

f. Brachytherapy 10%

g. Abstention

15. What is the best option for local recurrence after conservative treatment of the primary lesion?

a. Repeat conservative treatment 10%

b. Partial Amputation 87%

c. Total Amputation 3%

d. Radiotherapy 0%

e. Abstention

16. What is the best way to treat T2 tumors confined to the spongious body/glans?

a. Local excision/glansectomy with reconstruction 13%

b. Partial Amputation 75%

c. Total Amputation 2%

d. Isolated radiotherapy 5%

e. Chemo-radiotherapy 5%

f. Abstention

17. What is the best way to treat T2/T3 tumors invading the corpora cavernosa/urethra?

a. Isolated radiotherapy 0%

b. Partial Amputation 56%

c. Total Amputation 41%

d. Chemo-radiotherapy 3%

e. Abstention

18. What is the best way to treat T4 tumors invading neighboring structures?

a. Total Amputation/Emasculation 51%

b. Neoadjuvant chemotherapy followed by surgery if there is a response 49%

c. Isolated radiotherapy 0%

d. Chemo-radiotherapy 0%

e. Abstention

19. What is the best treatment for local recurrence after radical treatment of the primary lesion?

a. Partial Reamputation 18%

b. Total Amputation 62%

c. Chemotherapy 13%

d. Radiotherapy 7%

e. Abstention

# **treatment of locally advanced disease**

20. Early inguinal lymphadenectomy (LI) has better 5-year survival rates (compared with late LI) but should be indicated only for patients at high risk of lymph-node involvement.

a. Yes 82%

b. No 18%

c. Abstention

21. Radical LI should always be bilateral due to presymphyseal lymphatic crossover and studies showing more than 50% bilateral inguinal drainage:

a. Yes 90%

b. No 10%

c. Abstention

22. Guided aspiration biopsy of suspected inguinal lymph nodes, guided by imaging methods, has not been unequivocally confirmed as a safe method of lymph-node involvement. Therefore:

a. Not to be recommended 27%

b. Should always be recommended 2%

c. May aid in diagnosis and is not routinely recommended 71%

d. Abstention

23. The main prognostic factors for inguinal lymph-node metastases used to indicate IL are: ≥pT1b stage, microscopic lymphatic invasion, perineural invasion, invasion >0.5 mm and palpable lymph nodes, after primary tumor resection and appropriate antibiotic therapy:

a. Yes 98%

b. No 2%

c. Abstention

24. High immunohistochemical expression of p53, MMP 2 and MMP9, and low Ki-67 expression, the infiltrating growth pattern rather than growth by displacement (“pushing”) and unfavorable histology (usual, basaloid, adenosquamous, mixed and sarcomatoid squamous-cell carcinoma) are variables associated, in several studies, with higher risk of regional lymph-node metastases. Should these characteristics always be taken into account in the therapeutic indication of IL?

a. Yes 30%

b. No 9%

c. Can be used whenever possible 61%

d. Abstention

25. The social factor, configured by the uncertainty of adherence to follow-up after treatment of the primary tumor, may be an indication of IL, comparing risks and benefits:

a. Yes 46%

b. No 8%

c. Only in individualized cases 46%

d. Abstention

26. Low risk factors for inguinal lymph-node involvement and contributing to the contraindication of LI: Intraepithelial neoplasms (*in situ*), pT1a stage, papillary or Warty tumors, with non-invasive vertical exophytic growth:

a. Yes 98%

b. No 2%

c. Abstention

27. Sentinel lymph-node dynamic biopsy (BDLS): Regarding pelvic lymph-node involvement and its treatment:

a. Is always indicated 0%

b. Is never indicated by limitations of logistics, experience and reproducibility 21%

c. Should be performed only at referral centers with trained staff 79%

d. Abstention

28. Should inguinal lymphadenectomy (in open, laparoscopic or robotic technique) be bilateral, except if BDLS is negative on one side?

a. Yes 69%

b. No 31%

c. Abstention

29. Fixed, stony or unresectable lymph nodes should undergo neoadjuvant chemotherapy. For patients not responsive to chemotherapy or contraindicated, the palliative options are:

a. Inguinal lymphadenectomy 4%

b. Radiotherapy 46%

c. Options “a” and “b” 50%

d. Abstention

30. When only one inguinal lymph node is affected without extracapsular extension and <4.0cm can active surveillance be recommended?

a. Yes 64%

b. No 36%

c. Abstention

31. If two or more inguinal lymph nodes are affected, or in the presence of extracapsular extension, is pelvic lymphadenectomy always indicated?

a. Yes 81%

b. No 19%

c. Abstention

32. Pelvic lymph-node involvement is usually ipsilateral to inguinal involvement. Is it acceptable to perform unilateral ipsilateral LP?

a. Yes 70%

b. No 30%

c. Abstention

# **surgical aspects**

33. In locally unresectable disease with involvement of the posterior urethra and or prostate, what type of urinary diversion/reconstruction should be avoided?

a. Nephrostomy 0%

b. Cystostomy 6%

c. Mitrofanoff 6%

d. Ileal conduit 0%

e. Perineal urethrostomy 88%

f. Abstention

34. Multimodal therapy has proven to be a good option for treating advanced disease in penile cancer. The best surgical treatment option for urinary tract reconstruction/shunt in disseminated metastatic disease is:

a. Mitrofanoff 0%

b. Ileal conduit 15%

c. Cystectomy + Bricker 5%

d. Cystostomy 70%

e. Palliative care without intervention 10%

f. Abstention

35. Multimodal therapy has been shown to be a good option for the treatment of advanced disease in penile cancer, aiming at a reduction in tumor volume. In these cases, which of the surgical treatment options for urinary tract reconstruction/shunt, aiming for a better quality of life, would you avoid performing?

a. Mitrofanoff 6%

b. Ileal conduit 0%

c. Cystectomy + ileal conduit 61%

d. Cystostomy 11%

e. Palliative care without intervention 22%

f. Abstention

36. In the case of surgical resection failure, in which the tumor was resectable, the next approach will be:

a. Immediately perform urinary diversion 8%

b. Refer to endovascular 0%

c. Chemotherapy 41%

d. Radiotherapy 5%

e. Chemoradiotherapy 46%

f. Refer for palliative care 0%

g. Abstention

37. What is the best approach after tumor-mass reduction by chemotherapy?

a. Surgical approach 92%

b. Radiotherapy 4%

c. Chemotherapy 2%

d. Urinary Bypass 0%

e. Second Line Chemo 2%

f. Palliative Care 0%

g. Abstention

38. Should we still discuss Hemi Pelvectomy and/or Hemi Corporectomy?

a. Yes 35%

b. No 65%

c. Abstention

# **Unresectable or relapsed tumors**

39. Local recurrence infiltrating the perineum without lymph-node involvement. What is the recommendation?

a. Surgical approach with possibility of colostomy and cystostomy 26%

b. Neoadjuvant chemotherapy to re-approach if possible 69%

c. Definitive palliative chemotherapy 6%

d. Radiotherapy 0%

e. Palliative Care 0%

f. Abstention

40. Advanced local recurrence with inguinal unilateral or bilateral lymph-node mass without vascular involvement. What is the recommendation?

a. Surgical re-approach with possibility of colostomy and cystostomy + lymphadenectomy 8%

b. Neoadjuvant chemotherapy to re-approach if possible 92%

c. Definitive palliative chemotherapy 0%

d. Radiotherapy 0%

e. Palliative Care 0%

f. Abstention

41. Advanced local recurrence with unilateral or bilateral fixed inguinal lymph-node mass with vascular involvement. What is the recommendation?

a. Surgical re-approach with possibility of colostomy and cystostomy + lymphadenectomy + femoral lame disarticulation or vascular bypass 3%

b. Neoadjuvant chemotherapy to re-approach if possible 77%

c. Definitive palliative chemotherapy 20%

d. Radiotherapy 0%

e. Palliative Care 0%

f. Abstention

42. Local recurrence or advanced tumor without inguinal lymph-node involvement with visceral metastasis. What is the recommendation?

a. Surgical approach with possibility of colostomy and cystostomy + lymphadenectomy + metastasis resection 5%

b. Definitive palliative chemotherapy 92%

c. Radiotherapy 0%

d. Palliative Care 3%

e. Abstention

43. Advanced local recurrence with unilateral or bilateral fixed inguinal lymph-node mass with vascular involvement and visceral metastasis. What is the recommendation?

a. Surgical approach with the possibility of colostomy and cystostomy + lymphadenectomy + femoral lame disarticulation + resection of metastases 2%

b. Definitive palliative chemotherapy 90%

c. Radiotherapy 0%

d. Palliative Care 8%

e. Abstention

44. Locally treated and controlled disease, with visceral recurrence. What is the recommendation?

a. Surgical treatment, metastasis resection 7%

b. Definitive palliative chemotherapy 93%

c. Radiotherapy 0%

d. Embolization 0%

e. Abstention

45. Local recurrence after chemotherapy/radiotherapy without prior surgical approach. What is the recommendation?

a. Surgical approach with possibility of colostomy and cystostomy 77%

b. Definitive palliative chemotherapy 15%

c. Radiotherapy 0%

d. Palliative Care 8%

e. Abstention

46. Visceral recurrence after local surgical resection, chemo and/or radiotherapy. What is the recommendation?

a. Surgical resection of metastasis 7%

b. Definitive palliative chemotherapy 81%

c. Palliative Care 12%

d. Abstention

47. Local and visceral recurrence after surgical resection, first- and second- line chemo and/or radiotherapy, what is the recommendation?

a. Surgical re-approach with possibility of colostomy and cystostomy + Metastasis resection 7%

b. Embolization of pelvic vessels 0%

c. Exclusive embolization of metastasis 0%

d. Palliative Care 93%

e. Abstention

# **radiotherapy**

48. When can treatment aimed at preserving the penis through radiotherapy be recommended in penile squamous cell carcinoma?

a. Patient preference and/or inoperable for comorbidities 15%

b. T1 or T2 tumors 5%

c. Tumors less than 4cm 5%

d. All above 72%

e. Always 0%

f. Never 3%

g. Abstention

49. Patients undergoing radical radiotherapy should undergo prostectomy:

a. Whenever possible 90%

b. Rarely 10%

c. Never 0%

d. Abstention

50. For patients with T1 G1-2 tumors smaller than 4cm who choose radical radiotherapy, concomitance with chemotherapy is indicated:

a. Never 77%

b. Always 23%

c. Abstention

51. Contact brachytherapy for tumors up to 5mm deep is an acceptable radical-purpose radiotherapy technique in patients with T1 G1-2 tumors smaller than 4cm:

a. Yes 100%

b. No 0%

c. Abstention

52. Interstitial brachytherapy is an acceptable radical-purpose radiotherapy technique in patients with T1 G1-2 tumors smaller than 4cm:

a. Yes 97%

b. No 3%

c. Abstention

53. Teletherapy is an acceptable radical-purpose radiotherapy technique in patients with T1 G1-2 tumors smaller than 4cm:

a. Yes 88%

b. No 12%

c. Abstention

54. In radical-purpose teletherapy in patients with T1 G1-2 tumors smaller than 4cm, elective irradiation of inguinal and pelvic lymph nodes is indicated:

a. Always 0%

b. Never 100%

c. Abstention

55. For patients with T1 G3-4 or T2 tumors smaller than 4cm, who choose radiotherapy with radical purpose, concomitance with chemotherapy is indicated:

a. Never 47%

b. Always 53%

c. Abstention

56. Interstitial brachytherapy is an acceptable radical-purpose radiotherapy technique in patients with T1 G3-4 and T2 tumors smaller than 4cm:

a. Yes 70%

b. No 30%

c. Abstention

57. Teletherapy is an acceptable radical-purpose radiotherapy technique in patients with T1 G3-4 and T2 tumors smaller than 4cm:

a. Yes 85%

b. No 15%

c. Abstention

58. In radical-purpose teletherapy in patients with T1 G3-4 and T2 tumors smaller than 4cm, elective irradiation of inguinal and pelvic lymph nodes is indicated:

a. Always 38%

b. Never 62%

c. Abstention

59. For patients with T1-T2 tumors larger than 4cm who choose radical radiotherapy, concomitance with chemotherapy is indicated:

a. Never 30%

b. Always 70%

c. Abstention

60. Interstitial brachytherapy is an acceptable radical-purpose radiotherapy technique in patients with T1-T2 tumors larger than 4cm:

a. Yes 35%

b. No 65%

c. Abstention

61. Teletherapy is an acceptable radical-purpose radiotherapy technique in patients with T1-T2 tumors larger than 4cm:

a. Yes 55%

b. No 45%

c. Abstention

62.Teletherapy + interstitial brachytherapy dose increment is an acceptable radical-purpose radiotherapy technique in patients with T1-T2 tumors >4cm:

a. Yes 65%

b. No 35%

c. Abstention

63. In radiotherapy with radical purpose in patients with T1-T2 tumors >4cm, elective irradiation of inguinal and pelvic lymph nodes is indicated:

a. Always 54%

b. Never 46%

c. Abstention

64. For patients with T3-T4 tumors who receive radical-purpose radiotherapy, concomitance with chemotherapy is indicated:

a. Never 15%

b. Always 85%

c. Abstention

65. Interstitial brachytherapy is an acceptable radical-purpose radiotherapy technique in patients with T3-T4 tumors:

a. Yes 6%

b. No 94%

c. Abstention

66. Teletherapy is an acceptable radical-purpose radiotherapy technique in patients with T3-T4 tumors:

a. Yes 47%

b. No 53%

c. Abstention

67. Teletherapy + interstitial brachytherapy dose enhancement is an acceptable radical-purpose radiotherapy technique in patients with T3-T4 tumors:

a. Yes 43%

b. No 57%

c. Abstention

68. In radical-purpose teletherapy in patients with T3-T4 tumors, elective irradiation of inguinal and pelvic lymph nodes is indicated:

a. Always 68%

b. Never 32%

c. Abstention

69. When radical teletherapy (with or without chemotherapy) is indicated in which lymph nodes will not be treated, what is the minimum technique for administering the radiation dose?

a. Modulated Intensity Radiotherapy (IMRT) 21%

b. Conformational Radiotherapy (RT3D) 58%

c. Conventional Radiotherapy (R2D) 21%

d. Abstention

70. When there is indication of radical teletherapy (with or without chemotherapy) in which the lymph nodes will not be treated, what is the ideal technique for the administration of radiation dose?

a. Modulated Intensity Radiotherapy (IMRT) 7%

b. Imaging-guided radiotherapy (IGRT) 7%

c. Conformational Radiotherapy (RT3D) 7%

d. Conventional Radiotherapy (R2D) 0%

e. Combination of options “a” and “b” 57%

f. Combination of options “b” and “c” 7%

g. Options “a” or “c” 15%

h. Abstention

71. When there is indication of radical teletherapy (with or without chemotherapy) in which the lymph nodes will be treated, what is the minimum technique for radiation dose administration?

a. Modulated Intensity Radiotherapy (IMRT) 11%

b. Conformational Radiotherapy (RT3D) 84%

c. Conventional Radiotherapy (R2D) 5%

d. Abstention

72. When there is indication of radical teletherapy (with or without chemotherapy) in which the lymph nodes will be treated, what is the ideal technique for the administration of radiation dose?

a. Modulated Intensity Radiotherapy (IMRT) 6%

b. Imaging-guided radiotherapy (IGRT) 6%

c. Conformational Radiotherapy (RT3D) 6%

d. Conventional Radiotherapy (R2D) 0%

e. Combination of options “a” and “b” 69%

f. Combination of options “b” and “c” 0%

g. Options “a” or “c” 13%

h. Abstention

73. Is a compromised surgical margin an indication of adjuvant radiotherapy in penile cancer?

a. Yes 60%

b. No 40%

c. Abstention

74. Is pT3-pT4N0 with free margins an indication of adjuvant radiotherapy in penile cancer?

a. Yes 5%

b. No 95%

c. Abstention

75. Should patients with cN2 disease not receiving neoadjuvant treatment and undergoing inguinal and pelvic lymph-node dissection receive adjuvant radiotherapy?

a. Yes, if ≥pN2 45%

b. Yes, only if pN3 18%

c. No, just follow up despite lymph-node status after surgery 37%

d. Abstention

# **locally advanced disease**

76. What should be the immediate course in patients with unilateral mobile unilateral lymph-node enlargement ≥4cm with biopsy-confirmed lymph-node metastatic disease?

a. Inguinal lymph-node dissection 60%

b. Neoadjuvant chemotherapy followed by inguinal lymph-node dissection 40%

c. Abstention

77. Should patients with unilateral mobile lymph-node enlargement ≥4cm undergoing neoadjuvant chemotherapy who have residual disease in one or more inguinal lymph-nodes undergo pelvic lymphadenectomy?

a. Yes 86%

b. No 14%

c. Abstention

78. What should be the initial approach for patients with bilateral or fixed inguinal lymph-node enlargement without pelvic lymph-node enlargement on imaging?

a. Neoadjuvant chemotherapy 62%

b. Primary tumor resection + inguinal lymphadenectomy 19%

c. Primary tumor resection + inguinal and pelvic lymphadenectomy 19%

d. Abstention

79. For patients with resected pelvic lymph-node enlargement identified on staging imaging, what is the best immediate approach?

a. Primary tumor resection + inguinal and pelvic lymphadenectomy 77%

b. Neoadjuvant chemotherapy 23%

c. Chemotherapy + radiotherapy 0%

d. Abstention

80. For patients with unresectable pelvic lymph-node enlargement identified on staging imaging, what is the best immediate approach?

a. Neoadjuvant chemotherapy (conversion chemotherapy) 76%

b. Chemotherapy + radiotherapy 12%

c. Palliative intensive chemotherapy 12%

d. Abstention

81. For patients with resectable cN3 disease who do not have complete or partial clinical response to neoadjuvant chemotherapy but have no disease progression during chemotherapy, what should be the next approach?

a. Primary tumor resection + inguinal and pelvic lymphadenectomy 92%

b. Second line chemotherapy with different treatment regimen 4%

c. Combined radiotherapy + chemotherapy treatment 4%

d. Abstention

82. Should patients with cN2 or cN3 disease with clinical response to neoadjuvant chemotherapy with ypN + residual disease receive adjuvant treatment?

a. Radiotherapy 44%

b. Chemotherapy 4%

c. Radiotherapy + chemotherapy 20%

d. Follow Only 32%

e. Abstention

83. What is the most appropriate neoadjuvant chemotherapy regimen when indicated?

a. Cisplatin based double scheme 8%

b. Cisplatin based triple scheme (with taxane) 92%

c. Scheme without platinum (Vinblastine + Bleomycin + Methotrexate) 0%

d. Abstention

84. The most suitable imaging modality for locoregional response evaluation to neoadjuvant chemotherapy is:

a. Pelvic magnetic nuclear resonance (MRI) 20%

b. Pelvic Computed Tomography (CT) 17%

c. MRI or pelvic CT 53%

d. PET-CT with 18-FDG 10%

e. Abstention

85. The most suitable imaging modality for distance disease assessment during/after neoadjuvant chemotherapy is:

a. CT scan of upper abdomen and chest 83%

b. Upper abdominal CT and chest X-ray 7%

c. Upper abdominal ultrasound (US) and chest X-ray 0%

d. PET-CT with 18-FDG 10%

e. Abstention

86. Should patients that underwent primary tumor resection and inguinal lymphadenectomy without receiving neoadjuvant treatment with pN1 disease receive adjuvant treatment?

a. Yes, only chemotherapy 41%

b. Yes, only radiotherapy 6%

c. Yes, radiotherapy + chemotherapy 19%

d. No, just follow up 34%

e. Abstention

87. Should patients not undergoing neoadjuvant treatment but who had inguinal and pelvic lymphadenectomy and have metastases in pelvic lymph nodes, with bilateral inguinal lymph-node involvement or extranodal extension receive adjuvant treatment?

a. Yes, chemotherapy 38%

b. Yes, radiotherapy 6%

c. Yes, chemotherapy + radiotherapy 53%

d. No, just follow up 3%

e. Abstention

88. What is the most appropriate adjuvant chemotherapy regimen when indicated?

a. Cisplatin based double scheme 27%

b. Cisplatin based triple scheme (with taxane) 67%

c. Scheme without platinum (vinblastine + bleomycin + methotrexate) 6%

d. Abstention

# **Relapsed disease without possibility of local rescue/metastatic DISEASE**

89. In first-line therapy for unresectable, recurrent and/or metastatic penile cancer, what is the standard therapy?

a. Cisplatin and Fluorouracil 11%

b. Cisplatin/Fluorouracil/Docetaxel (TPF) 37%

c. Cisplatin/Ifosfamide/Paclitaxel (TIP) 52%

d. Cisplatin/Methotrexate/Bleomycin (CMB) 0%

e. Abstention

90. Which option to choose for second-line chemotherapy after TIP/TPF failure in unresectable, relapsed and/or metastatic cancer?

a. Paclitaxel monotherapy 16%

b. Cisplatin/Irinotecan 28%

c. Anti-EGFR target therapy (Cetuximab/Panitumumab) +/- chemotherapy 36%

d. Exclusive Clinical Support 20%

e. Abstention

91. What is the role of anti-EGFR targeted therapy in the current treatment of advanced penile cancer?

a. Must be used 7%

b. May be recommended based on results from other tumors 34%

c. Investigational 59%

d. Abstention

# **follow up**

92. In patients treated for early penile cancer (N0), what tests are needed to follow up an asymptomatic patient?

a. Anamnesis + Clinical Examination 27%

b. Anamnesis + Clinical examination + US + Rx 38%

c. Anamnesis + Clinical examination + CT + MRI 35%

d. Anamnesis + Clinical examination + CT + MRI + bone scintigraphy 0%

e. Abstention

93. If previous patient imaging (N0) is required, how often are they requested?

a. Every 3 months for the first 2 years, every 6 months for the fifth year and then annually 28%

b. Every 3 months for the first 2 years, every 6 months for the fifth year 34%

c. Every 3 months in the first year, every 6 months until the fifth year 23%

d. Every 3 months until the fifth year 0%

e. Every 3 months until the second year 0%

f. Every 3 months in the first year and 6 months in the second year 3%

g. Every six months until the second year 6%

h. Every six months until the fifth year 6%

i. Abstention

94. Patient treated for penile cancer and compromised lymph node (N1), which tests are necessary to follow the asymptomatic patient?

a. Anamnesis + Clinical Examination 3%

b. Anamnesis + Clinical examination + US + Rx 23%

c. Anamnesis + Clinical examination + CT + MRI 71%

d. Anamnesis + Clinical examination + CT + MRI + bone scintigraphy 3%

e. Abstention

95. If imaging is required in the above patient (N1), how often are they requested?

a. Every 3 months for the first 2 years, every 6 months for the fifth year and then annually 33%

b. Every 3 months for the first 2 years, every 6 months for the fifth year 47%

c. Every 3 months in the first year, every 6 months until the fifth year 14%

d. Every 3 months until the fifth year 0%

e. Every 3 months until the second year 0%

f. Every 3 months in the first year and 6 months in the second year 3%

g. Every six months until the second year 3%

h. Every six months until the fifth year 0%

i. Abstention

96. Patient treated for penile cancer and compromised lymph node (N2-3), which exams are necessary to follow the asymptomatic patient?

a. Anamnesis + Clinical Examination 5%

b. Anamnesis + Clinical examination + US + Rx 5%

c. Anamnesis + Clinical examination + CT + MRI 83%

d. Anamnesis + Clinical examination + CT + MRI + bone scintigraphy 7%

e. Abstention

97. If patient imaging is required above (N2-3), how often is this requested?

a. Every 3 months for the first 2 years, every 6 months for the fifth year and then annually 31%

b. Every 3 months for the first 2 years, every 6 months for the fifth year 56%

c. Every 3 months in the first year, every 6 months until the fifth year 2%

d. Every 3 months until the fifth year 0%

e. Every 3 months until the second year 0%

f. Every 3 months in the first year and 6 months in the second year 3%

g. Every six months until the second year 3%

h. Every six months until the fifth year 5%

i. Abstention

98. How is follow-up of advanced penile tumors (unresectable/inoperable) established?

a. Physical examination every 3 months 0%

b. Physical and imaging exam every 6 months 3%

c. Examination of images every 3 months 15%

d. Physical examination and imaging each year 3%

e. Individual approach with referral to palliative care as needed 79%

f. Abstention

99. When should the patient be referred for psychological support?

a. Always, since the diagnosis 96%

b. Only in unresectable/metastatic disease 2%

c. In most cases 0%

d. In the minority of cases 0%

e. Never 2%

f. Abstention

# **bone therapy**

100. For patients with bone metastases, would you indicate bone-modifying agents (consider patients without clinical contraindications, eg, allergies, kidney failure, etc.)?

a. For all patients 89%

b. Never 11%

c. Abstention

101. Among the available bone-modifying agents, do you have a preference for:

a. Zoledronic acid 21%

b. Denosumab 38%

c. No preference 41%

d. Abstention

102. For patients using zoledronic acid, the dose and frequency you recommend are:

a. 4mg IV every 4 weeks 57%

b. 4mg IV every 12 weeks 43%

c. Abstention

103. For patients using denosumab, the dose and frequency you recommend are:

a. 120mg SC every 4 weeks 84%

b. 120mg SC every 12 weeks 16%

c. Abstention

104. For patients with malignant penile neoplasia with bone metastases, the duration of therapy with bone modifying agents should be:

a. No time limit or until a significant/intolerable adverse event 75%

b. Up to 24 months 22%

c. Until a new bone event 3%

d. Abstention
